# Metabolic Profiling of Cognitive Aging in Midlife

**DOI:** 10.3389/fnagi.2020.555850

**Published:** 2020-11-05

**Authors:** Zhiguang Huo, Brinda K. Rana, Jeremy A. Elman, Ruocheng Dong, Corinne D. Engelman, Sterling C. Johnson, Michael J. Lyons, Carol E. Franz, William S. Kremen, Jinying Zhao

**Affiliations:** ^1^Department of Biostatistics, University of Florida, Gainesville, FL, United States; ^2^Department of Psychiatry, University of California, San Diego, San Diego, CA, United States; ^3^Population Health Sciences, University of Wisconsin School of Medicine and Public Health, Madison, WI, United States; ^4^Wisconsin Alzheimer’s Institute, University of Wisconsin School of Medicine and Public Health, Madison, WI, United States; ^5^Alzheimer’s Disease Research Center, University of Wisconsin School of Medicine and Public Health, Madison, WI, United States; ^6^Department of Psychological & Brain Sciences, Boston University, Boston, MA, United States; ^7^Center of Excellence for Stress and Mental Health, Veterans Affairs San Diego Healthcare System, San Diego, CA, United States; ^8^Department of Epidemiology, University of Florida, Gainesville, FL, United States

**Keywords:** Alzheimer’s disease, untargeted metabolomics, cognitive change, middle age, twin studies

## Abstract

Alzheimer’s dementia (AD) begins many years before its clinical symptoms. Metabolic dysfunction represents a core feature of AD and cognitive impairment, but few metabolomic studies have focused on cognitive aging in midlife. Using an untargeted metabolomics approach, we identified metabolic predictors of cognitive aging in midlife using fasting plasma sample from 30 middle-aged (mean age 57.2), male-male twin pairs enrolled in the Vietnam Era Twin Study of Aging (VETSA). For all twin pairs, one twin developed incident MCI, whereas his co-twin brother remained to be cognitively normal during an average 5.5-year follow-up. Linear mixed model was used to identify metabolites predictive of MCI conversion or cognitive change over time, adjusting for traditional risk factors. Results from twins were replicated in an independent cohort of middle-aged adults (mean age 59.1) in the Wisconsin Registry for Alzheimer’s Prevention (WRAP). Results in twins showed that higher baseline levels of four plasma metabolites, including sphingomyelin (d18:1/20:1 and d18:2/20:0), sphingomyelin (d18:1/22:1, d18:2/22:0, and d16:1/24:1), DAG (18:2/20:4), and hydroxy-CMPF, were significantly associated with a slower decrease in one or more domains of cognitive function. The association of sphingomyelin (d18:1/20:1 and d18:2/20:0) was replicated in WRAP. Our results support that metabolic perturbation occurs many years before cognitive impairment and plasma metabolites may serve as early biomarkers for prediction or monitoring of cognitive aging and AD in midlife.

## Introduction

Human brain aging and Alzheimer’s dementia (AD) impose a huge health and economic burden on modern society. Recent research suggests that cognitive decline begins in middle age (45–65 years old) (Singh-Manoux et al., 2012; Karlamangla et al., 2017). However, there is a substantial heterogeneity in the trajectories of cognitive aging, with some individuals showing minimal or no decline while others of the same age experiencing rapid decline or even dementia. Discovery of sensitive, specific and non-invasive biomarkers is a prerequisite for identifying high-risk individuals, and developing effective strategies that can monitor, delay or prevent the onset of dementia.

A growing body of evidence suggests that brain and cognitive aging are accompanied by extensive metabolic perturbations (Clarke et al., 2018). The altered metabolic profiles can be quantified by metabolomics, a high-throughput biochemical technology that can identify hundreds to thousands of small molecules (metabolites) in biofluids or tissues (Rochfort, 2005). These metabolites represent the endpoints of metabolic processes encompassing the interaction between the internal genome and the external environment (Harrigan and Goodacre, 2012), and thus are closer to disease phenotypes compared to genomic, transcriptomic and proteomic profiles.

While the majority of the existing metabolomics studies in human (Han et al., 2011; Orešič et al., 2011; Björkqvist et al., 2012; Mapstone et al., 2014; Inoue et al., 2015; Casanova et al., 2016; Varma et al., 2018; Huo et al., 2020) have focused on older individuals (aged 65+), several recent studies reported metabolic changes associated with cognitive aging (Bressler et al., 2017; Chouraki et al., 2017; Darst et al., 2018; van der Lee et al., 2018; Proitsi et al., 2018; Tynkkynen et al., 2018) among individuals in midlife (45–65 years). However, most of these studies have examined a small number of metabolites (in general less than 300), leaving the full spectrum of blood metabolome largely unexplored. To date, no consensus metabolites that can predict brain and cognitive aging in midlife have been reported.

Leveraging the comprehensive clinical and cognitive phenotypes in a well-characterized longitudinal twin cohort, the current study aimed to identify metabolites predictive of incident mild cognitive impairment (MCI) or cognitive change using untargeted metabolomics in 30 middle-aged (mean age 57.2 at baseline), male-male twin pairs [15 monozygotic (MZ) pairs, 15 dizygotic (DZ) pairs]. All twins were cognitively normal at baseline (Wave 1, 2003–2007), but one twin in each of these pairs developed incident MCI, while his co-twin remained cognitively normal at the end of 5.5-year follow-up (Wave 2, 2008–2013). Our initial study was followed by replication in an independent cohort of middle age adults (mean age 59.1 at blood draw). Although preliminary, findings of this research are likely to generate novel hypotheses that may lead to the identification of novel metabolic markers predictive of cognitive aging in midlife and provide insights into our understanding of the role of metabolic disturbance in early cognitive aging.

## Materials and Methods

### Study Populations

#### Discovery Cohort – Vietnam Era Twin Study of Aging (VETSA)

Our discovery sample included 30 middle-aged, male-male twin pairs (15 MZ pairs and 15 DZ pairs) participating in the VETSA, a longitudinal observational study examining the role of genetic and environmental factors for cognitive and brain aging beginning in midlife using a twin design. Detailed methods for twin recruitment, longitudinal follow-up and phenotypes for the twins had been described previously (Kremen et al., 2006, 2013a). Briefly, 1,237 twins (349 MZ pairs, 265 DZ pairs, 9 singletons, age range 51–60, mean age 56) attended the initial examination at Wave 1 (2003–2007), and 1,016 twins were re-examined at Wave 2 (2008–2013, mean follow-up 5.5 years). The only two recruitment criteria for the VETSA were: (1) the ages of participants at Wave 1 were between 51 and 59 years at the time of initial recruitment (2003–2007), and (2) both twins in a pair were willing to participate in the baseline assessment. Twins enrolled in VETSA were predominately Caucasians (86%). Although all twins served in the United States military between 1965 and 1975, nearly 80% of them did not serve in combat or Vietnam (Kremen et al., 2006, 2011). Since 25% of the men nationwide within this age range served in the military, the VETSA participants were generally representative of middle-aged men living across the United States, with respect to health and sociodemographic characteristics (Schoenborn and Heyman, 2009). Participants were administered identical protocols at the University of California, San Diego, or Boston University. Individual twins chose their site, although brothers most often chose the same site. The complete protocols for twin enrollment and exams had been described previously (Kremen et al., 2013a). The protocols were approved by the University of California and Boston University Institutional Review Boards.

The current analysis included 30 complete pairs (mean age 57.2, age range 52.6–59.6, all Caucasians) attending the clinical exam at Wave 1 (baseline, 2003–2007) followed through Wave 2 (2008–2013). All twins were cognitively normal at baseline, but one twin per pair developed incident MCI by Wave 2 (mean follow-up period 5.5 years) while their co-twin did not. Both twins of the same pair were examined at the same site. The baseline characteristics of these twins are shown in Table 1. There was no significant difference in the listed characteristics at baseline between twins who converted to MCI (converters) versus their co-twins who did not (non-converters).

**TABLE 1:** Baseline characteristics of twins in the VETSA (*N* = 60).

Characteristics		Mean±*SD*or%		*P*-value*
	
	All	MCI non-converter	MCI converter	
*N*	60	30	30	
Mean age (year) at baseline	57.22.3	57.22.3	57.22.3	0.97
Mean age (year) at Wave 2	62.82.3	62.72.3	62.82.3	0.97
BMI (kg/m^2^)	304.2	304.6	303.7	0.49
Education (year)	142	142.1	141.9	0.53
Ever-smoker, *n* (%)**^†^**	23 (38)	13 (43)	10 (33)	0.60
General cognitive ability	0.280.66	0.320.69	0.250.65	0.677
Episodic memory	−0.0720.72	0.00460.74	−0.150.7	0.414
Short term memory	−0.230.66	−0.110.71	−0.360.58	0.133
Executive function	−0.130.27	−0.0680.24	−0.190.29	0.071

*P-values comparing non-converters and converters were calculated using t-test for continuous variables, and Fisher’s exact test for binary variables. ^†^Defined as participants who smoked 100 cigarettes or more in lifetime.

#### Replication Cohort – Wisconsin Registry for Alzheimer’s Prevention (WRAP)

We replicated our initial findings in the WRAP (La Rue et al., 2008), which is an ongoing prospective cohort that enrolls middle-aged adults and examines the transition from middle age to early older adults (between 40 and 65). Participants had no dementia at baseline. The WRAP allows for the enrollment of siblings and is enriched for a parental history of Alzheimer’s disease. Detailed information for the study design and methods has been described elsewhere (Sager et al., 2005; Johnson et al., 2018). The current analysis included 232 WRAP participants (mean age 59.1, age range 40–72, 32% males, all Caucasians) with complete clinical and metabolomics data at baseline (2011–2015) and follow-up (average follow-up period = 4.1 years). Supplementary Table 1 displays the baseline characteristics of the WRAP participants.

### Cognitive Phenotypes and Diagnostics

#### Vietnam Era Twin Study of Aging (VETSA)

All twins enrolled in the VETSA underwent extensive neurocognitive testing including 13 neuropsychological tests (23 scores) covering 7 cognitive domains (Kremen et al., 2019) that were designed to avoid ceiling effects in middle-age adults. Specifically, general cognitive ability was measured by the Armed Forces Qualification Test (AFQT), a 50-min paper-and-pencil test with 100 multiple-choice items on vocabulary, arithmetic, spatial processing, and mechanical ability (Uhlaner and Bolanovich, 1952; Orme et al., 2001). The AFQT is highly correlated (∼0.85) with other measures of general cognitive ability, including the Wechsler scales of intelligence (Lyons et al., 2009, 2017). Executive function was assessed by a factor comprising 7 measures of prepotent response inhibition, task-set shifting, and working memory: Stroop interference; AX-Continuous Performance Test; Delis-Kaplan Executive System (D-KEFS) Trails switching; D-KEFS category switching; Wechsler Memory Scale-III (WMS-III) Letter-number sequencing; Reading span; and WMS-III Digit span (Gustavson et al., 2018). Episodic memory was measured by summarizing verbal and visual-spatial episodic memory measures, compromising 6 individual episodic memory measures (Kremen et al., 2014), including California Verbal Learning Test-II short/long delayed free recall; WMS-III logical memory immediate/delayed free recall, and visual reproductions immediate/delayed recall. Short-term memory was derived based on standardized and averaged scores from WMS-III digit span (forward condition) and spatial span (forward condition) (Franz et al., 2011). MCI status was determined via the Jak-Bondi approach, requiring at least 2 tests within a cognitive domain to each be more than 1.5 SDs below normative expectations in order to define impairment in that domain (Jak et al., 2009; Kremen et al., 2013b; Granholm et al., 2017).

#### WRAP

As previously described (Dowling et al., 2010; Koscik et al., 2014), the WRAP has also collected detailed neurocognitive phenotypes for all participants. Since the WRAP did not have the AFQT, we used the average of verbal and visuospatial ability from the Wechsler Abbreviated Scale of Intelligence (Koscik et al., 2014) as a surrogate measure for the general cognitive ability. Executive function in the WRAP was calculated by averaging the *z*-score of the following four components (Clark et al., 2016), including trail making test part B (TMT B) total time to completion; Stroop neuropsychological screening test color-word interference total items completed in 120 s; Wechsler abbreviated intelligence scale-revised (WAIS-R); and digit symbol coding total items completed in 90 s. We used a composite of delayed recall scores (Clark et al., 2016) as a surrogate measure for episodic memory: Rey Auditory Verbal Learning Test long-delay free recall, WMS-R logical memory delayed recall, Brief Visuospatial Memory Test-Revised delayed recall. Since WRAP did not collect short-term memory, we used immediate memory, assessed by a factor compromising Trials 1 and 2 of Rey Auditory Verbal Learning Test (Schmidt, 1996), in our replication analysis.

### Metabolomics Data Acquisition

Fasting blood samples were drawn in the morning of the same day when cognitive tests were performed. Plasma samples were collected (EDTA tube), aliquoted and then stored at −80°C. Untargeted metabolomics data in fasting plasma samples were assayed in both VETSA (*n* = 60) and WRAP (*n* = 232) using the same protocols on the same platform at Metabolon Inc. Detailed procedures for metabolomics analysis have been described elsewhere (Evans et al., 2009, 2014). Briefly, automated MicroLab STAR^®^ system from Hamilton Company was used to remove protein and recover chemically diverse metabolites. The consequent extract was equally aliquoted into five parts for different analyses, including two separate reverse phase (RP)/UPLC-MS/MS methods for positive ion mode electrospray ionization (ESI), one RP/UPLC-MS/MS method for negative ion mode ESI, one HILIC/UPLC-MS/MS method for negative ion mode ESI, and the remaining one for backup purposes. Quality control samples were also included to ensure the Metabolon procedures were conducted within the specified protocol. Internal standards (endogenous compounds) were spiked into each analyzed sample to monitor instrument performance and aid chromatographic alignment. Positive and negative samples were randomized and QC samples were evenly spaced among the injections. After drying, the samples were reconstituted according to the pre-mentioned methods, followed by utilization of Ultrahigh Performance Liquid Chromatography-Tandem Mass Spectroscopy (UPLC-MS/MS). Metabolon’s software was used to extract the raw data and perform peak-identification, QC processing, and biochemical identification. A series of curation procedures were performed to guarantee the quality of the data. The relative intensity of each metabolite was quantified using area-under-the curve (AUC) of its corresponding peak.

### Metabolomics Data Pre-processing and Quality Control

For the 30 twin pairs in VETSA, we obtained a total of 1,228 metabolites, including 960 known and 268 unknown compounds. The AUCs of all samples were first normalized to their extracted volume, followed by median normalization (average of the median levels in all samples) as previously described (van den Berg et al., 2006). The normalized data were further log2 transformed to improve normality. Metabolites with missing values in >50% of the samples were removed. Metabolites with missing value ≤ 50% of the samples were replaced by limit of detection (LOD). A total of 1,131 metabolites (879 knowns and 252 unknowns) was included in the final analysis in the VETSA. Similar procedures were used to pre-process and QC the metabolomics data in the WRAP (Darst et al., 2019), resulting in a total of 1,097 metabolites in our replication analysis.

### Statistical Analyses

#### Identifying Metabolites Predictive of MCI Onset and Cognitive Change in VETSA

##### Discordant twin analyses

To identify plasma metabolites that can predict incident MCI, we first performed paired t-tests by comparing the relative abundance of metabolites between MCI converters and non-converters. These discordant twin analyses can be particularly powerful because twins within pairs are nearly perfectly matched for demographic, developmental, and environmental factors, resulting in considerably reduced error variance compared with non-twin studies. In addition, MZ pairs are perfectly matched for genetic background, and DZ pairs share, on average, 50% of their genetic background.

##### Non-twin analyses

While matched pair analysis is powerful, it does not allow for adjustments of other covariates. Thus, we also conducted non-twin analyses, meaning that the unit of analysis was the individual rather than the twin pair. This was done by constructing generalized linear mixed models via the *R* package *lme4* (Bates et al., 2014), in which MCI status (y/n) was the dependent variable, and baseline metabolite level was the independent variable, adjusting for twin age, BMI, smoking, and education at baseline and zygosity (MZ vs. DZ). To identify metabolites that can predict the longitudinal change in cognitive function over time, we fitted linear mixed models, in which change in a cognitive measure (difference in a cognitive measure between Wave 1 and Wave 2) was the dependent variable and baseline metabolite level was the independent variable, adjusting for twin age, zygosity (MZ vs. DZ), education, BMI, smoking, and the cognitive measure at baseline. The effect size and *P*-value were estimated for each metabolite for each cognitive measure. In the above described models, twin pair was included as a random effect to adjust for the correlated observations. Multiple testing was controlled by false discovery rate (FDR) (Benjamini and Hochberg, 1995) (adjusting for a total of 1,228 metabolites × 5 cognitive phenotypes including MCI, general cognitive ability, executive function, episodic memory, and short-term memory), and FDR-adjusted *P*-value was used to determine statistical significance.

#### Replication in the WRAP

To replicate the metabolites identified in the VETSA, we fitted linear mixed models in the WRAP, in which cognitive change (difference in a cognitive measure between baseline and follow-up) was the dependent variable and baseline plasma level was the independent variable. The model adjusted for age, sex, education, BMI, and the cognitive measure under investigation at baseline. Family ID was included as a random effect in the model to account for relatedness between family members.

##### Network analysis

To identify metabolic networks (modules) associated with cognitive functions, we conducted the weighted gene correlation network analysis (WGCNA) (Langfelder and Horvath, 2008) using all 1,131 metabolites (both known and unknown) in the VETSA. The structure of each module was constructed using data from MCI non-converters (*n* = 30) via the *ARACNE* algorithm (Margolin et al., 2006) using *R* package *minet* (Meyer et al., 2008). To examine whether module structure predicts incident MCI, we performed modular differential connectivity (MDC) analysis (McKenzie et al., 2016) by comparing the network connectivity between MCI converters and non-converters. The statistical significance of the MDC analysis was assessed by 1,000 permutations. The network structure of the modules containing significant MDC was visualized by *CytoScape* (Shannon et al., 2003).

##### Functional annotation

To functionally annotate the metabolites predictive of MCI conversion or cognitive change over time, we conducted pathway enrichment analysis using the software *MetaboAnalyst* 4.0 (Chong et al., 2019). Metabolites with raw *P* < 0.05 were used as input, and hypergeometric test was employed to obtain *P*-values for metabolic pathways.

## Results

### Metabolites Predictive of Incident MCI or Cognitive Change in VETSA

**FIGURE 1:**
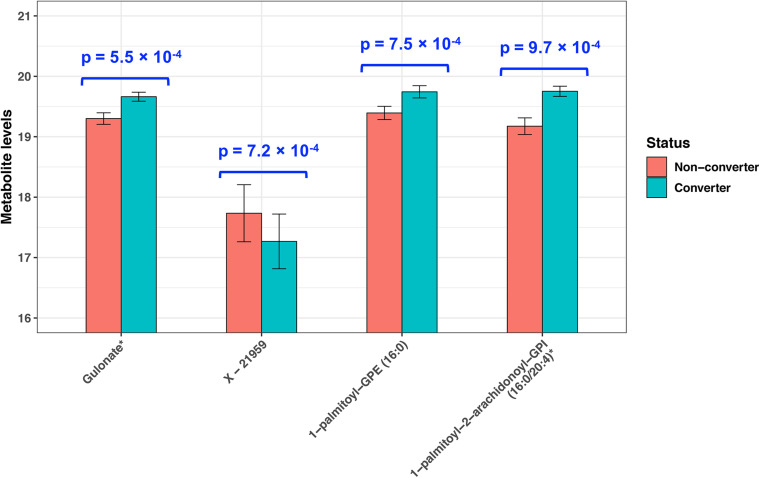
Plasma metabolites predictive of incident MCI in twins (*P* < 0.001). *P*-values were obtained by paired *t*-test.

At raw *P* < 0.001, we identified four plasma metabolites that can predict MCI conversion (Figure 1). Specifically, higher levels of three known metabolites [gulonate, β = 0.36, *P* = 5.5 × 10^–4^; 1-palmitoyl-GPE (16:0), β = 0.35, *P* = 7.5 × 10^–4^; and 1-palmitoyl-2-arachidonoyl-GPI (16:0/20:4) (16:0/20:4), β = 0.58, *P* = 9.7 × 10^–4^] and a lower level of one unknown metabolite (*X* − 21959, β = −0.47, *P* = 7.2 × 10^–4^) predicted MCI conversion. These P-values remained similar after adjustments for age, cognition, zygosity, education, BMI, and smoking status at baseline (Supplementary Table 2). However, none of them passed multiple comparison at FDR-adjusted *P* < 0.05.

**FIGURE 2:**
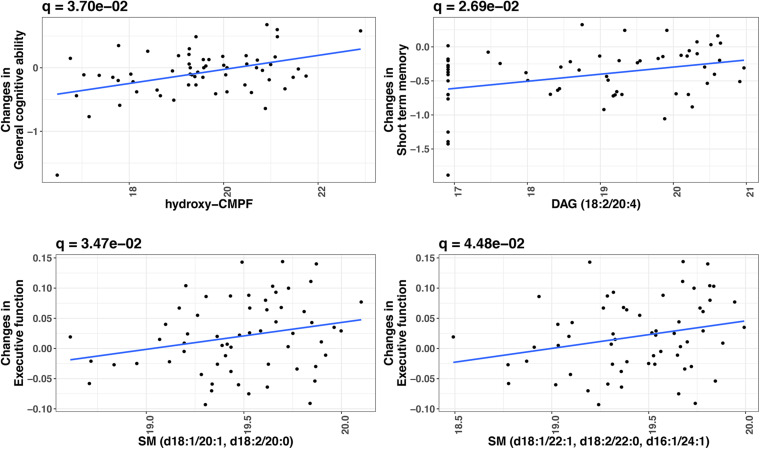
Significant metabolites predictive of cognitive change over time in twins (FDR-adjusted *P* < 0.05). *X*-axis indicates the relative abundance in plasma metabolite (log2 transformed) at baseline. *Y*-axis indicates cognition change over time (Wave 1 to Wave 2). *P*-values were obtained by linear mixed models, in which change in a cognitive measure (difference in a cognitive measure between Wave 1 and Wave 2) was the dependent variable and metabolite level was the independent variable, adjusting for twin age, zygosity (MZ vs. DZ), education, BMI, smoking, and the cognitive measure at baseline. Twin pair was included as a random effect in the model.

At FDR-adjusted *P* < 0.05, we found that baseline levels of four plasma metabolites significantly predicted longitudinal change in cognitive function over time (Table 2). Specifically, higher baseline levels of hydroxy-CMPF, linoleoyl-arachidonoyl-glycerol [DAG] (18:2/20:4), sphingomyelin [SM] (d18:1/20:1 and d18:2/20:0), and SM (d18:1/22:1, d18:2/22:0, and d16:1/24:1) significantly predicted a slower decrease in general cognitive ability (β = 0.16, *q* = 0.04), short-term memory (β = 0.16, *q* = 0.03), and executive function (β = 0.04, *q* = 0.03;β = 0.04, *q* = 0.04), respectively. Figure 2 depicts the associations of these metabolites with cognitive functions. No metabolites significantly predicted changes in episodic memory at FDR-adjusted *P* < 0.05.

**TABLE 2:** Plasma metabolites whose baseline level predict cognitive changes over time in twins (*q*-value < 0.05).

Metabolite	Cognitive phenotype	Effect size*	*P*-value	*q*-value
Hydroxy-CMPF	General cognitive ability	0.16	2.60 × 10^–5^	0.04
DAG (18:2/20:4)	Short term working memory	0.16	4.75 × 10^–6^	0.03
SM (d18:1/20:1, d18:2/20:0)	Executive function	0.04	1.23 × 10^–5^	0.03
SM (d18:1/22:1, d18:2/22:0, d16:1/24:1)	Executive function	0.04	3.96 × 10^–5^	0.04

*Effect size indicates the effect of per log2 fold change in metabolite level on change in cognitive change over time.

### Replication in the WRAP

Of the four metabolites predictive of cognitive change in the VETSA, we were able to replicate the association of SM (d18:1/20:1 and d18:2/20:0) (*P* = 0.003) with longitudinal change in executive function in the WRAP. The other 3 metabolites showed consistent effect size direction, but statistically non-significant.

### Metabolic Networks

**FIGURE 3:**
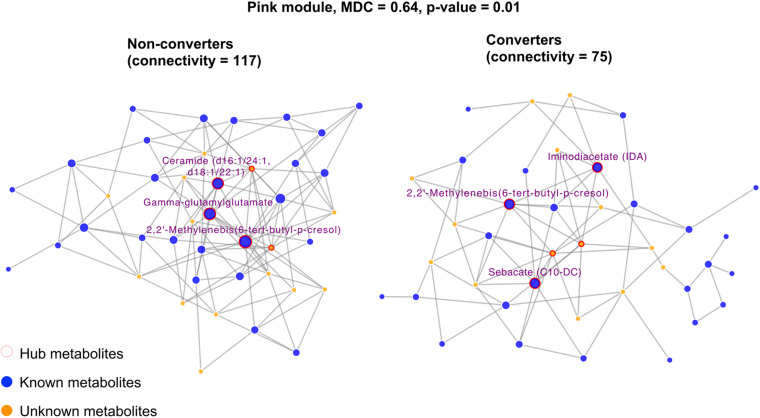
Differential network analysis showing significant structural changes of the pink module by comparing twins who developed incident MCI versus their co-twin brothers who did not. Only hub metabolites were labeled. The hub metabolites for NCI network were 2,2′-Methylenebis(6-tert-butyl-*p*-cresol), ceramide (d16:1/24:1, d18:1/22:1), and gamma-glutamylglutamate. The hub metabolites for the MCI network were 2,2′-Methylenebis(6-tert-butyl-p-cresol), iminodiacetate (IDA), and sebacate (C10-DC).

We identified 16 metabolite modules (Supplementary Figure 1 and Supplementary Table 3). Of these, the network connectivity (a measure quantifying the connections among metabolites) for the pink module (Figure 3) was significantly higher in MCI non-converters compared to that in converters (117 vs. 75, MDC = 0.64, *p* = 0.01), suggesting that reduced connectivity between metabolites may be associated with MCI onset. In addition, we identified several hub metabolites that differ significantly between MCI converters and non-converters. For example, 2,2′-methylenebis(6-tert-butyl-p-cresol), ceramide (d16:1/24:1, d18:1/22:1) and gamma-glutamylglutamate were the hub metabolites only in non-converters but not in converters.

### Functional Annotation

The aminoacyl-tRNA biosynthesis pathway showed the most significant association with short-term memory (*P* = 4.59 × 10^–6^), followed by the sphingolipid metabolism pathway associated with MCI (*P* = 1.56 × 10^–4^), and the valine, leucine, and isoleucine biosynthesis pathway in relation to short-term memory (*P* = 5.02 × 10^–4^). The top five metabolic pathways are shown in Table 3.

**TABLE 3:** Top metabolic pathways derived using plasma metabolites predictive of MCI onset or cognitive change over time.

Metabolic pathway	MCI	GCA	EpiMem	STMem	EF
Aminoacyl-tRNA biosynthesis	5.02 × 10^–1^	2.26 × 10^–1^	1.47 × 10^–1^	4.59 × 10^–6^	6.38 × 10^–1^
Sphingolipid metabolism	1.56 × 10^–4^	3.29 × 10^–1^	2.61 × 10^–1^	3.74 × 10^–1^	8.26 × 10^–3^
Valine, leucine and isoleucine biosynthesis				5.02 × 10^–4^	
Nicotinate and nicotinamide metabolism		2.31 × 10^–3^	1.81 × 10^–2^		
Phenylalanine, tyrosine and tryptophan biosynthesis				2.73 × 10^–3^	

*MCI, mild cognitive impairment; GCA, general cognitive ability; EpiMem, episodic memory; STMem, short-term memory; EF, executive function.*

## Discussion

In an untargeted metabolomic analysis including 30 middle-aged twin pairs discordant for progression to MCI, we identified four fast plasma metabolites whose baselines significantly predict cognitive change over time after adjusting for multiple testing and known covariates. Of these, the association between a sphingomyelin (SM [d18:1/20:1, d18:2/20:0]) and the longitudinal change in executive function was replicated in an independent cohort of middle-aged adults. Metabolic network analysis revealed that a reduced metabolic connection could be associated with progression to MCI. The identified metabolites were enriched in several metabolic pathways. Together, our results provide initial evidence that metabolic perturbation affects cognitive aging in midlife and suggest that plasma metabolites may serve as potential biomarkers for early cognitive aging and AD prediction in midlife.

Of the four metabolites predictive of cognitive change identified in twins, higher baseline levels of two sphingomyelins predict a slower decline in executive function, and one of these associations was replicated in WRAP. This finding is in agreement with findings from previous epidemiologic study among older adults (age 65+) (Mielke et al., 2010, 2011; Han et al., 2011; Orešič et al., 2011). The relationship between sphingomyelins and cognitive function was also consistent with previous research demonstrating that a disrupted sphingomyelin pathway is involved in Aβ pathology (Jana and Pahan, 2004), and a reduced level of sphingomyelin in human AD brain than that in cognitively healthy individuals (He et al., 2010). We were unable to replicate the other three metabolites in the WRAP, probably due to the small sample size, the heterogeneous clinical phenotypes across different studies, or other differences such as WRAP included women as well and participants who were enriched with a parental history of AD. The lack of replication could also be attributed to false positives as a result of the small sample size in our discovery cohort.

We also found a higher baseline level of plasma DAG (18:2/20:4) was associated with a slower decrease in short-term memory. While the precise mechanism underlying this association is unclear and awaits further investigation, the association appears to be consistent with previous studies showing that neuronal glycerolipid biosynthesis promotes axon regeneration after optic nerve injuries (Yang et al., 2020), and that diffuse axonal injury causes cognitive impairment including short-term memory (Wallesch et al., 2001). In addition, a higher plasma level of hydroxy-CMPF was associated with a slower decrease in general cognitive ability. The CMPF is a furan fatty acid that inhibits insulin secretion in both human and mouse models (Nagy et al., 2017). A higher level of CMPF has been associated with type 2 diabetes (Prentice et al., 2014) and biological aging (Menni et al., 2013) in human. While these findings are seemingly contradictory to the protective effect of hydroxy-CMPF on cognitive function identified in the current study, it is possible that hydroxylation of CMPF may reduce the plasma concentration of CMPF, which could likely improve neuronal insulin resistance, thereby exerting beneficial effects on cognition.

Our network analysis revealed that loss of metabolites connectivity (the pink module) may be related to cognitive change, and we identified several hub metabolites that differ significantly between MCI converters and non-converters. Although the precise mechanisms are unclear, it is likely that the differences in network connectivity and hub metabolites may explain, at least in part, why some twins progressed to MCI while his co-twin brother did not. For example, we found that 2,2′-methylenebis(6-tert-butyl-p-cresol), ceramide (d16:1/24:1, d18:1/22:1), and gamma-glutamylglutamate were the hub metabolites only in non-converters but not in converters. Ceramides are sphingolipids that play important roles in cellular processes including differentiation, proliferation, and apoptosis (Hannun and Obeid, 2008). Dysregulation of ceramides in the brain has been implied in Alzheimer’s and other neurodegenerative disease (Filippov et al., 2012; Jazvinšćak Jembrek et al., 2015). Gamma-glutamylglutamate acid is a dipeptide consisting of gamma-glutamate and glutamic acid. Glutamic acid is the main excitatory neurotransmitter in the brain, which can be released to neurons and form glutamate; the latter can convert to glutamic acid via astrocytes, which completes the glutamate-glutamine cycle (Daikhin and Yudkoff, 2000). Dysregulation of glutamate signaling has been implicated in neurodegeneration and cognitive behaviors (Vogel et al., 1966; Rahn et al., 2012).

Among the metabolic pathways we identified to be associated with cognitive changes, the aminoacyl-tRNA biosynthesis pathway showed the most significant association with changes in short-term memory. The process of aminoacyl tRNA biosynthesis catalyzes the ligation of amino acids to their cognate tRNAs, and dysregulation of this pathway has been associated with neurodegeneration (Park et al., 2008; Kapur et al., 2017). The observed association of the amino acid (valine, leucine, and isoleucine) pathway with short-term memory is consistent with previous research demonstrating that branched-chain amino acids (BCAAs) were associated with dementia (Tynkkynen et al., 2018) and metabolic conditions such as insulin resistance and diabetes (Roberts et al., 2014), both of which have been linked to cognitive impairment (Ma et al., 2015) and AD (Arnold et al., 2018).

In this study, we identified associations of circulating plasma metabolites with cognitive change over time, independent of known risk factors. The mechanisms underlying the observed associations are unclear but believed to be highly complex. While genome-wide association studies (GWAS) have identified many genetic variants associated with cognitive functions, cognitive impairment and AD in various human populations under different clinical settings (Trampush et al., 2017; Jansen et al., 2019), the identified genetic variants explain only a small proportion of the disease risk. A larger contributor to the phenotypic variation may be due to the complex interactions between environmental and inherited factors. Metabolomics provides a snapshot of hundreds to thousands of small molecules (metabolites) in a biological sample (e.g., plasma) at a given time. The abundance of these metabolites is influenced by both genetic and environmental factors, such as diet, lifestyle, behavior, drug, microbiome, hormones, etc., probably through various epigenetic mechanisms. Metabolomic study of circulating metabolites could facilitate the identification of environmental factors contributing to cognition changes during aging, and provides mechanistic insight into how environmental exposures contribute to cognitive impairment in AD progression. Moreover, we identified that different plasma lipids were associated with different cognitive domains, including general cognitive ability, short term memory, executive memory, but not episodic memory. While the mechanism underlying this differential associations remain to be determined, the lack of association with episodic memory might suggest that the underlying pathological mechanisms are dementia-related but not necessarily specific to AD. However, it is worth noting that the VETSA and WRAP samples are both relatively young. Although prospective studies of AD usually emphasize episodic memory, there is also evidence suggesting that executive function can predict progression to MCI or AD as early or possibly earlier than memory deficits (Clark et al., 2012; Ewers et al., 2012; Gibbons et al., 2012). Executive function deficits, which were associated with metabolites in the present study, are also common in MCI and AD, and executive control functions do affect functioning in other cognitive domains (Buckner, 2004; Baudic et al., 2006). Indeed, there is also evidence for an executive-prominent AD subtype (Mukherjee et al., 2012). Thus, although it remains uncertain, it is possible that the associated deficits that were observed will contribute to AD-related deficits.

Limitations of our study include the small sample size and the inclusion of only Caucasian men in the twin study, the sample differences across studies such as the selection of WRAP biased toward parental history of AD, and the different cognitive measures in our discovery and replication cohorts. The uncertainty of the unknown metabolites represents another limitation. However, our study has several strengths, including a focus on midlife, the well-matched MCI discordant twin pairs (which eliminates confounding by age and sex and minimized confounding by multiple background factors), the inclusion of a replication cohort comprised of middle-aged adults in a similar age range, the prospective design of both discovery and replication cohorts, the comprehensive cognitive phenotypes, and the comprehensive coverage of plasma metabolome by using an untargeted metabolomics approach on the same platform in both cohorts.

## Conclusion

In summary, in an untargeted metabolomics analysis of middle-aged adults in two well characterized prospective cohorts, we identified four plasma metabolites that are significantly predictive of cognitive change over time, after adjusting for multiple testing and known clinical covariates. The observed association of a sphingomyelin (SM [d18:1/20:1, d18:2/20:0]) with executive function was replicated in an independent cohort including participants in a similar age range. We also identified metabolic networks and hub metabolites that may be involved in the relationship between metabolic dysregulation and cognitive impairment in midlife. Our results support that metabolic perturbation occurs many years before cognitive impairment, and suggest the possibility of using blood metabolites as non-invasive biomarkers in early prediction of cognitive aging and AD in midlife.

## Data Availability Statement

The data analyzed in this study is subject to the following licenses/restrictions: The authors will accept reasonable requests for data access, which will be referred to the Veteran Era Twin Registry where the data are maintained and overseen. Requests to access these datasets should be directed to http://www.vetsatwins.org.

## Ethics Statement

The studies involving human participants were reviewed and approved by twins participating in the VETSA were administered identical protocols at the University of California, San Diego, or Boston University. Participants in the WRAP were administered at the University of Wisconsin. Written consents were obtained from all participants enrolled in both studies. The protocols were approved by Institutional Review Boards at the participating universities. The patients/participants provided their written informed consent to participate in this study.

## Author Contributions

ZH, BR, and JE performed the statistical analysis. RD, CE, and SJ performed the replication analysis in WRAP. ML, CF, WK, and JZ designed and coordinated the study. ZH and JZ wrote the manuscript. All authors reviewed the manuscript.

## Conflict of Interest

The authors declare that the research was conducted in the absence of any commercial or financial relationships that could be construed as a potential conflict of interest. The reviewer LY declared a past co-authorship with several of the authors ZH and JZ to the handling editor.
