# Supplementary material for: Metabolic Profiling of Cognitive Aging in Midlife
Source: Front Aging Neurosci. 2020 Nov 5;12:555850. doi: 10.3389/fnagi.2020.555850 (PMC7674168; doi:10.3389/fnagi.2020.555850)
Supplement: Supplementary file 1 [file Table_1.DOCX]

**Table S1**. Baseline characteristics of the WRAP participants (n=232)

| Characteristics | Mean ± SD or % |
| --- | --- |
| Age (year) at baseline | 59.1 ± 7.0 |
| Gender, n (%) | 74 (31.9) |
| Mean follow up time | 4.1 ± 0.5 |
| BMI (kg/m^2^) | 28.7 ± 5.9 |
| Education (year) | 16.0 ± 2.3 |
| General cognitive ability | 0.16 ± 0.82 |
| Episodic memory | 0.14 ± 0.68 |
| Immediate memory | 0.05 ± 1.06 |
| Executive function | 0.07 ± 0.74 |

**Table S2.** Plasma metabolites predictive of incident MCI in twins.

| Metabolite | Abbreviation | Paired t test^*^ | | Linear mixed model^†^ | |
| --- | --- | --- | --- | --- | --- |
|  |  | Effect Size^‡^ | p-value | Effect Size | p-value |
| gulonate |  | 0.36 | 5.5×10^-4^ | 0.32 | 0.002 |
| X - 21959 |  | -0.47 | 7.2×10^-4^ | -0.49 | 0.001 |
| 1-palmitoyl-GPE (16:0) | LysoPE (16:0/0:0) | 0.35 | 7.5×10^-4^ | 0.32 | 0.003 |
| 1-palmitoyl-2-arachidonoyl-GPI (16:0/20:4) | GPI(16:0/20:4) | 0.58 | 9.7×10^-4^ | 0.56 | 0.001 |

^*^ Paired t-test did not adjust for any covariates.

^†^ Linear mixed model adjusted for baseline age, zygosity (MD vs DZ), education, BMI, and smoking as covariates. Twin pair was included as a random effect in the model.

^‡^ Effect size indicates the log2 fold change in plasma metabolite level between twins who converted to MCI and his co-twin brother who remained to be cognitively normal by end of 6-year follow-up.

**Figure S1.** Metabolite membership for the co-regulated modules identified by WGCNA. Co-regulation dendrogram was obtained by clustering the dissimilarity based on topological overlap matrix distance. Highly co-regulated metabolites are represented by a unique color under the dendrogram, which is denoted by the module memberships. Sixteen modules were identified. Grey color indicates that metabolites did not fall into any module.

**Table S3.** Topology analysis showing the differential metabolites connectivity (MCI converters vs non-converters).

| Module | Size^*^ | Connectivity | | MDC^†^ | p-value^‡^ |
| --- | --- | --- | --- | --- | --- |
|  |  | Non-converters | Converters |  |  |
| Pink | 41 | 117 | 75 | 0.64 | 0.008 |
| Cyan | 25 | 44 | 29 | 0.67 | 0.054 |
| Green | 49 | 109 | 83 | 0.76 | 0.072 |
| Black | 44 | 105 | 81 | 0.77 | 0.094 |
| Salmon | 26 | 54 | 29 | 0.55 | 0.096 |
| Midnightblue | 22 | 45 | 30 | 0.67 | 0.118 |
| Lightcyan | 21 | 50 | 33 | 0.67 | 0.138 |
| Blue | 92 | 234 | 274 | 1.17 | 0.25 |
| Magenta | 40 | 92 | 107 | 1.16 | 0.276 |
| Yellow | 56 | 136 | 117 | 0.86 | 0.434 |
| Purple | 34 | 92 | 79 | 0.86 | 0.518 |
| Turquoise | 143 | 478 | 450 | 0.94 | 0.594 |
| Tan | 28 | 51 | 55 | 1.08 | 0.614 |
| Brown | 82 | 293 | 274 | 0.94 | 0.626 |
| Red | 49 | 111 | 115 | 1.04 | 0.76 |
| Greenyellow | 28 | 57 | 52 | 0.91 | 0.762 |

^*^ Size indicates the number of metabolites in a module.

^†^ MDC: modular differential connectivity, defined as (connectivity MCI + 1)/(connectivity NCI + 1).

^‡^ Two-sided p-value quantifying the significance level of MDC.
